# Adverse events of tumor necrosis factor alpha inhibitors for the treatment of ankylosing spondylitis: A meta-analysis of randomized, placebo-controlled trials

**DOI:** 10.3389/fphar.2023.1084614

**Published:** 2023-02-13

**Authors:** Haihuan Feng, Ying Zhao, Weihong Kuang, Yanping Dai, Xiaobo Cen, Feng Qin

**Affiliations:** ^1^ State Key Laboratory of Biotherapy, Collaborative Innovation Center for Biotherapy, National Chengdu Center for Safety Evaluation of Drugs, West China Hospital, Sichuan University, Chengdu, China; ^2^ Medical Insurance Office, West China Hospital, Sichuan University, Chengdu, China; ^3^ Department of Psychiatry, West China Hospital, Sichuan University, Chengdu, China; ^4^ Andrology Laboratory, Department of Urology, West China Hospital, Sichuan University, Chengdu, China

**Keywords:** ankylosing spondylitis, tumor necrosis factor alpha inhibitors, randomized controlled trial, adverse events, infection

## Abstract

**Objective:** Tumor necrosis factor alpha inhibitors (TNFi) have shown substantial efficacy in alleviating and treating ankylosing spondylitis (AS). However, the heightened interest is accompanied by concerns over adverse events. In this meta-analysis, we analyzed both serious and common adverse events in patients treated with tumor necrosis factor alpha inhibitors compared with those in the placebo group.

**Methods:** We searched for clinical trials in PubMed, Embase, Cochrane Library, China National Knowledge Infrastructure, Wanfang Data, and VIP Data. Studies were selected based on strict inclusion and exclusion criteria. Only randomized, placebo-controlled trials were included in the final analysis. RevMan 5.4 software was used for performing meta-analyses.

**Results:** A total of 18 randomized controlled trials recruiting 3,564 patients with ankylosing spondylitis were included, with overall moderate to high methodological quality. Compared with the placebo group, the incidences showed no difference and were only slightly increased numerically for serious adverse events, serious infections, upper respiratory tract infection, and malignancies in patients treated with tumor necrosis factor alpha inhibitors. However, tumor necrosis factor alpha inhibitor treatment significantly increased the incidence of overall adverse events, nasopharyngitis, headache, and injection-site reactions in ankylosing spondylitis patients when compared with placebo.

**Conclusion:** The available data indicated that ankylosing spondylitis patients who received tumor necrosis factor alpha inhibitors had no significantly increased risks of serious adverse events when compared with the placebo group. However, tumor necrosis factor alpha inhibitors significantly increased the incidence rate of common adverse events, including nasopharyngitis, headache, and injection-site reactions. Large-scale and long-term follow-up clinical trials are still necessary to further investigate the safety of tumor necrosis factor alpha inhibitors in ankylosing spondylitis treatment.

## 1 Introduction

Ankylosing spondylitis (AS) is a chronic inflammatory autoimmune disease that courses with the involvement of sacroiliac, axial, and peripheral joints (Zhu et al., 2019). The estimated global prevalence of AS ranges from 0.1 to 1.4% (Xiong et al., 2020). The prevalence of AS was 0.23% in the European population, 0.2%–0.5% in the United States, and 0.29% in China (Reveille, 2011; Dean et al., 2014; Zhao et al., 2020). AS is more common in young men aged 30–45 years with the characteristics of hidden onset, long course of the disease, and high disability rate, which cause a severe economic burden to patients and their families (Raychaudhuri and Deodhar, 2014; Xiong et al., 2020). The treatment for AS mainly includes non-steroidal anti-inflammatory drugs, tumor necrosis factor alpha inhibitors (TNFi), and interleukin-17A antagonists (Zhu et al., 2019; Shao et al., 2021).

As TNFi possess dramatic anti-inflammatory and immunomodulatory effects, they are widely used for the treatment of a range of inflammatory conditions, including rheumatoid arthritis, psoriatic arthritis, Crohn’s disease, and AS (Burmester et al., 2013). Currently, there are five commercially available TNFi for treating AS patients: adalimumab and golimumab, fully humanized anti-tumor necrosis factor (TNF) alpha monoclonal antibodies; infliximab, a chimeric murine–human full-length monoclonal antibody; etanercept, a fusion protein of human immunoglobulin G and two p75 TNF receptors; and certolizumab pegol, a humanized Fab fragment conjugated to polyethylene glycol (Mitoma et al., 2018). To date, the long-term use of TNFi in AS patients remains necessary, which raises many serious concerns regarding the safety of TNFi in AS patients (Wroński and Fiedor, 2019). Although some adverse events (i.e., malignancies, serious infections, and all-cause withdrawals) were observed among patients receiving TNFi, no significant association was noticed (Liu et al., 2016; Ma Z. et al., 2017; Xu et al., 2017; Hou et al., 2018). All meta-analyses on AS patients showed that there are no statistically significant differences in serious adverse events, serious infections, and malignancies between TNFi and placebo groups (Fouque-Aubert et al., 2010; Machado et al., 2013; Liu et al., 2016; Ma Z. et al., 2017; Xu et al., 2017; Hou et al., 2018). Nonetheless, there is no meta-analysis for assessing the risk of common adverse events, such as upper respiratory tract infection, nasopharyngitis, headache, and diarrhea.

This study aimed to analyze the available evidence of TNFi in AS treatment and conducted a meta-analysis by using the Cochrane system evaluation method. Specifically, the present study assessed the risks of both serious and common adverse events of TNFi in AS patients. The information would be useful to physicians for selecting the appropriate medications by considering the risk profile.

## 2 Materials and methods

### 2.1 Search strategy

To identify studies that reported the adverse events of TNFi in the treatment of AS, a systematic search in PubMed, Embase, Cochrane Library, China National Knowledge Infrastructure, Wanfang Data, and VIP Data was performed. Dates ranged from the inception of the different databases through 31 Aug 2022. The search terms were as follows: TNF, TNF-α, anti-TNF, tumor necrosis factor alpha, anti-TNF-alpha, etanercept, infliximab, adalimumab, golimumab, certolizumab pegol, and ankylosing spondylitis. We also searched for the references of the retrieved articles to identify additional studies. This literature review was conducted independently by two authors (HF and FQ), with a third resolving any disputes as needed (YZ). This meta-analysis had been conducted in accordance with the Preferred Reporting Items for Systematic Reviews and Meta-Analyses statement.

### 2.2 Inclusion criteria

Studies were included if they explicitly met the following criteria: 1) study design: the patients were randomly allocated to intervention groups (TNFi and placebo), and both parallel and crossover studies were included for eligibility; 2) population: the participants were patients with AS; 3) comparison: studies should contain the comparison of TNFi and the placebo; and 4) outcome: overall adverse events were used as the primary outcome, and serious adverse events, serious infections, upper respiratory tract infection, nasopharyngitis, malignancies, headache, diarrhea, and injection-site reactions were used as outcome indicators.

### 2.3 Exclusion criteria

Studies were excluded for meeting the following criteria: 1) case reports, animal studies, editorial comments, non-clinical outcome studies, and literature reviews; 2) unverified randomized controlled trials; 3) irrelevant outcomes; and 4) repeated articles or results. Two authors (HF and FQ) independently determined whether the studies met the inclusion criteria, with a third (YZ) resolving any disputes as needed.

### 2.4 Data extraction

For each included study, basic information and outcome indicators were extracted. Basic information relevant to this meta-analysis included: first author, year of publication, the country of the study, study design, sample size, age, gender, number of participants, duration of the follow-up, and intervention measures (the name and dosage of the medication and the type of placebo). Outcome indicators relevant to this meta-analysis included: overall adverse events, serious adverse events, serious infections, malignancies, upper respiratory tract infection, nasopharyngitis, injection-site reactions, headache, and diarrhea. The data were independently extracted by two authors (HF and FQ), with a third (YD) resolving any disputes as needed. If necessary, the reviewers would try to obtain incomplete information from the study investigators.

### 2.5 Bias assessment

The study quality was determined by using the Cochrane Collaboration bias risk tool, and the following factors were evaluated: 1) the study included a specific statement regarding randomization; 2) the method used to randomize patients was appropriate; 3) the study was conducted in a double-blinded manner; 4) the approach to double blinding was appropriately described; 5) information on any patients that withdrew from the study was provided; and 6) information on funding from the pharmaceutical companies.

### 2.6 Selected outcomes

A total of nine predefined outcomes were assessed. The primary outcome was the incidence of overall adverse events between TNFi and placebo groups in AS patients. The secondary outcomes were the incidence of serious adverse events, serious infections, upper respiratory tract infection, nasopharyngitis, injection-site reactions, malignancies, headache, and diarrhea between TNFi and placebo groups in AS patients.

### 2.7 Statistical analysis

RevMan 5.4 software (Cochrane Collaboration, London, United Kingdom) was used for all analyses. The risk of bias of the included studies was further evaluated by the Cochrane Collaboration’s tool. The proper effect sizes and statistical analysis methods were chosen according to different data types and evaluation purposes. For discontinuous outcomes, the odds ratio (OR) and 95% CI were calculated. We used fixed-effects models if there was no significant heterogeneity (I^2^ ≤ 50% or *p* > 0.1). Otherwise, we used random-effects models. The publication bias was assessed using the funnel plot.

## 3 Results

### 3.1 Literature search

**FIGURE 1:**
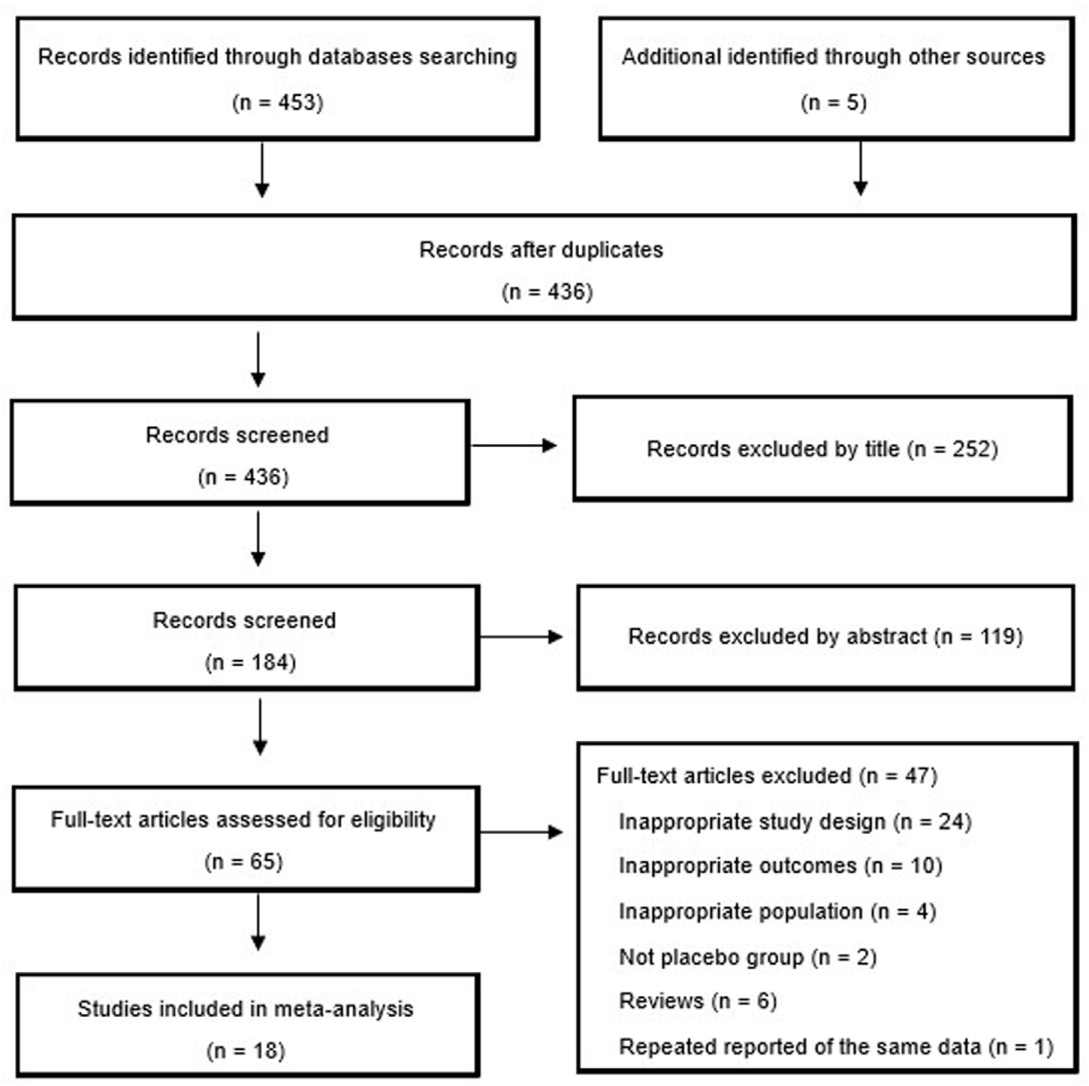
Study selection process for the meta-analysis with specifications of reasons.

The flow chart of the study selection process is presented in Figure 1. In total, 18 studies with 3,564 patients (2,282 in the TNFi group and 1,282 in the placebo group) were finally included in the present study. The studies were published between 2002 and 2022 and were primarily conducted in Europe (44.44%), North America (27.78%), and Asia (27.78%). All participants were AS patients. The main characteristics of the 18 studies are summarized in Table 1.

**TABLE 1:** Characteristics of the included studies in the meta-analysis.

Study	Country	Eligibility	Follow-up course	Number of cases		Age of the cases (Years)		Intervention measure		Outcome indicator
				TNFi	Control	TNFi	Control	TNFi	Control	
Gorman et al. (2002)	Germany	AS	16 weeks	20	20	38 ± 10	39 ± 10	Etanercept, 25 mg, twice weekly	Placebo	②④⑥⑨
Davis et al. (2003)	United States	AS	24 weeks	138	139	42.1	41.9	Etanercept, 25 mg, twice weekly	Placebo	②③④⑥⑧⑨
Calin et al. (2004)	United Kingdom	AS	12 weeks	45	39	45.3 ± 9.5	40.7 ± 11.4	Etanercept, 25 mg, twice weekly	Placebo	⑥⑧⑨
van der Heijde et al. (2006a)	Netherlands	AS	12 weeks	A: 155	51	A. 41.5 ± 11	40.1 ± 10.9	A. Etanercept, 50 mg, once weekly	Placebo	①②③④⑤⑥⑨
				B: 150		B. 39.8 ± 10.7		B. Etanercept, 25 mg, twice weekly		
Dougados et al. (2010)	France	AS	12 weeks	39	43	46 ± 11	48 ± 10	Etanercept, 50 mg, once weekly	Placebo	①②⑥⑦
Huang et al. (2010)	China	AS	6 weeks	74	78	30.4 ± 9.8	31.8 ± 9.5	Etanercept, 50 mg, once weekly	Placebo	①⑤⑥
Huang et al. (2011)	China	AS	6 weeks	300	100	29.1 ± 8.7	28.4 ± 8.0	Etanercept, 50 mg, once weekly	Placebo	①②⑦
van der Heijde et al. (2006b)	Netherlands	AS	24 weeks	208	107	41.7 ± 11.7	43.4 ± 11.3	Adalimumab, 40 mg, every other week	Placebo	①②③⑤⑥
Huang et al. (2014)	China	AS	12 weeks	229	115	30.1 ± 8.7	29.6 ± 7.5	Adalimumab, 40 mg, every other week	Placebo	①②③
van der Heijde et al. (2018)	Netherlands	AS	16 weeks	87	90	26.5 ± 8.6	26.4 ± 8.4	Adalimumab, 40 mg, every other week	Placebo	①②③④⑤⑥
Inman et al. (2008)	Canada	AS	24 weeks	A: 138	77	A: 30.0–47.0	31.0–50.0	A. Golimumab, 50 mg, every 4 weeks	Placebo	①②③④⑤⑥⑦⑧⑨
				B: 140		B: 29.0–46.0		B. Golimumab, 100 mg, every 4 weeks		
Bao et al. (2014)	China	AS	16 weeks	108	105	30.5 ± 10.27	30.6 ± 8.60	Golimumab, 50 mg, every 4 weeks	Placebo	①②③④⑥⑦
Ma Z. et al. (2017)	China	AS	24 weeks	13	12	28.2 ± 6.8	31.2 ± 5.6	Golimumab, 50 mg, every 4 weeks	Placebo	①④
Deodhar et al. (2018)	United States	AS	12 weeks	105	103	38.4 ± 10.1	39.2 ± 10.8	Golimumab, 2 mg/kg at 0, 4, and 12 weeks	Placebo	①②③⑨
Deodhar et al. (2022)	United States	AS	12 weeks	59	53	28–54	25–51	Golimumab, 2 mg/kg at 0, 4, and 12 weeks	Placebo	①②⑤⑥⑧
Braun et al. (2002)	Germany	AS	12 weeks	34	35	40.6 ± 8.0	39.0 ± 9.1	Infliximab, 5 mg/kg at 0, 2, and 6 weeks	Placebo	①②③④
van der Heijde et al. (2005)	Netherlands	AS	24 weeks	201	78	40.0	41.0	Infliximab, 5 mg/kg at 0, 2, 6, 12, and 18 weeks	Placebo	①②③④⑤⑥⑧⑨
Inman et al. (2010)	Canada	AS	12 weeks	39	37	42.9 ± 10.4	39.3 ± 9.0	Infliximab, 3 mg/kg at 0, 2, and 6 weeks	Placebo	①④⑤⑥⑧

Note: TNFi, tumor necrosis factor α inhibitors; AS, ankylosing spondylitis; ①, overall adverse event; ②, serious adverse event; ③, serious infection; ④, upper respiratory tract infection; ⑤, nasopharyngitis; ⑦, malignancy; ⑥, injection-site reaction; ⑧, headache; ⑨, diarrhea.

### 3.2 Methodological quality of the included studies

The methodological quality item for the 18 included studies is described in Supplementary Figure S1. Of these studies, one study did not state whether it was a double-blinded designed trial (Braun et al., 2002). Four studies conducted randomization using a web-based system (Inman et al., 2008; Huang et al., 2014; Ma H. et al., 2017; Deodhar et al., 2018), two studies using a randomized block methodology (van der Heijde et al., 2005; Bao et al., 2014), two studies using a randomization table (Huang et al., 2010; Huang et al., 2011), two studies using a computer-generated random sequence (Braun et al., 2002; van der Heijde et al., 2018), and the remaining studies provided unclear information about the random sequence generation. A total of 15 studies were funded by pharmaceutical companies, so they were marked with an unclear risk of bias for other biases.

### 3.3 Assessment of the primary outcome

**FIGURE 2:**
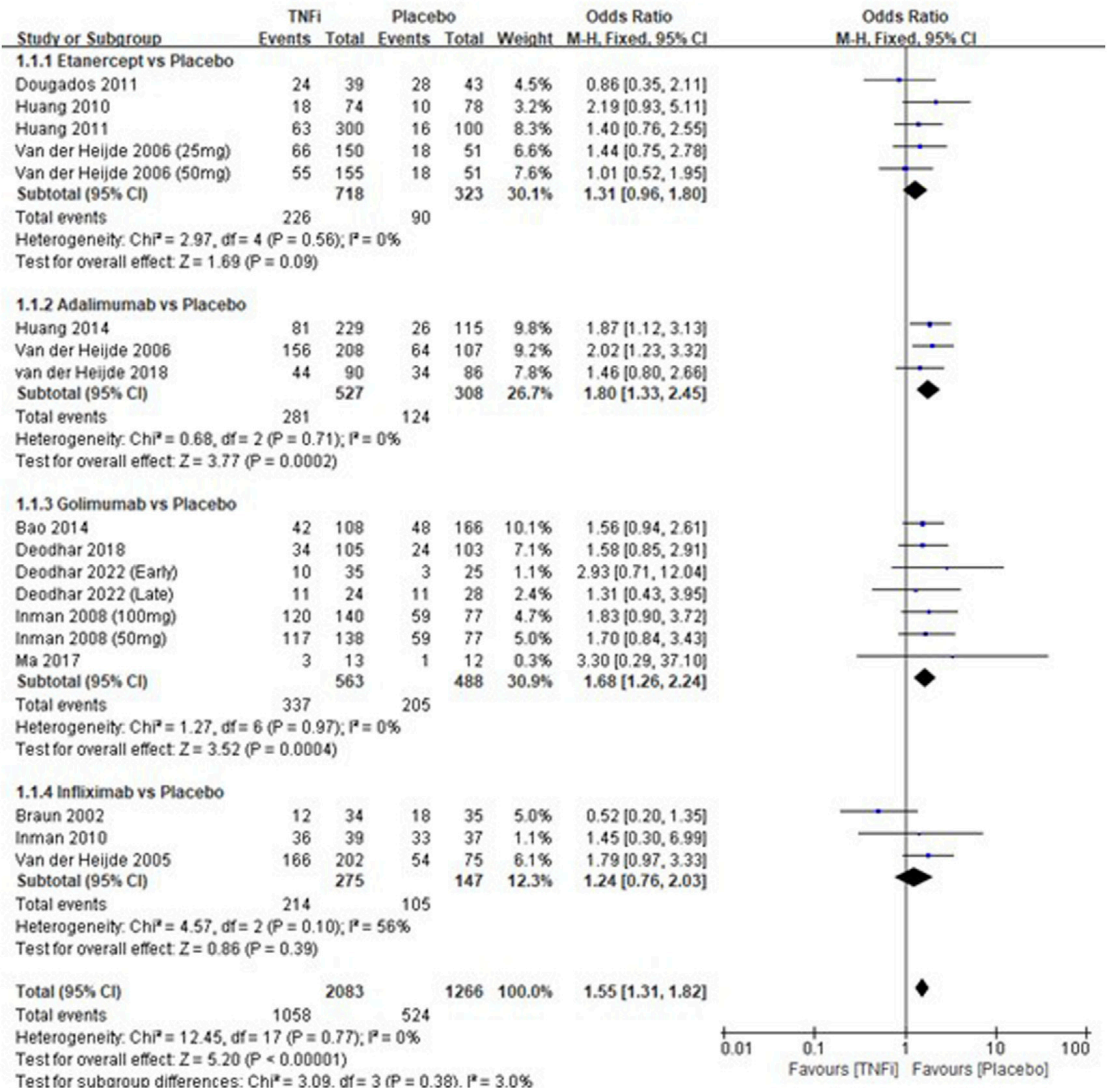
Pooled estimate of the incidence of overall adverse events between TNFi groups and placebo groups in patients with ankylosing spondylitis. The odds ratio >1.0 indicates that the incidence of serious adverse events is higher in the TNFi group than that in the placebo group. The subheading “Events” refers to the number of overall adverse events. “Total” refers to the total number of individuals. CI, confidence interval; df, degrees of freedom; M-H, Mantel–Haenszel method of calculation.

A total of 15 studies tested the incidence of overall adverse events between TNFi groups and placebo groups for AS treatment. As shown in Figure 2, a meta-analysis of the trials (*n* = 3,221) showed a significant increase in the incidence of overall adverse events for TNFi groups, compared to placebo groups (OR = 1.55, 95% CI: 1.31–1.82; *p* < 0.0001). The chi-squared test for homogeneity indicates that there were no statistical differences in the results among the trials (Chi^2^ = 12.45; df = 17; *p* = 0.77) with an I^2^ of 0% (I^2^ is typically considered low for <25%, modest for 25%–50%, and large for >50%), using the fixed-effects model. The subgroup results showed that there was a significant difference between adalimumab and placebo groups (OR = 1.80, 95% CI: 1.33–2.45; *p* = 0.0002) and between golimumab and placebo groups (OR = 1.68, 95% CI: 1.24–2.24; *p* = 0.0004), while no significant difference was found between etanercept and placebo groups (OR = 1.31, 95% CI: 0.96–1.80; *p* = 0.09) and between infliximab and placebo groups (OR = 1.24, 95% CI: 0.76–2.03; *p* = 0.39) in AS patients.

### 3.4 Assessment of the secondary outcome

A total of 12 studies tested the incidence of serious adverse events between TNFi groups and placebo groups for AS treatment. As shown in Supplementary Figure S2, a meta-analysis of the trials (*n* = 2,603) showed no significant difference in the incidence of serious adverse events between the two groups (OR = 1.37, 95% CI: 0.88–2.13; *p* = 0.17). The subgroup results showed that there was no significant difference in all the subgroups (etanercept *vs* placebo, adalimumab *vs* placebo, golimumab *vs* placebo, and infliximab *vs* placebo) in the incidences of serious adverse effects.

A total of 10 studies tested the incidence of serious infections between TNFi groups and placebo groups for AS treatment. As shown in Supplementary Figure S3, a meta-analysis of the trials (*n* = 2,590) showed no significant difference in the incidence of serious infections between the two groups (OR = 1.44, 95% CI: 0.66–3.16; *p* = 0.36). The subgroup results showed that there was no significant difference in all the subgroups (etanercept *vs* placebo, adalimumab *vs* placebo, golimumab *vs* placebo, and infliximab *vs* placebo) in the incidences of serious infections.

A total of 10 studies tested the incidence of upper respiratory tract infection between TNFi groups and placebo groups for AS treatment. As shown in Supplementary Figure S4, a meta-analysis of the trials (*n* = 1828) showed no significant difference in the incidence of upper respiratory tract infection between the two groups (OR = 1.22, 95% CI: 0.93–1.61; *p* = 0.16). The subgroup results also showed that there was no significant difference in all the subgroups (etanercept *vs* placebo, adalimumab *vs* placebo, golimumab *vs* placebo, and infliximab *vs* placebo) in the incidences of upper respiratory tract infection.

**FIGURE 3:**
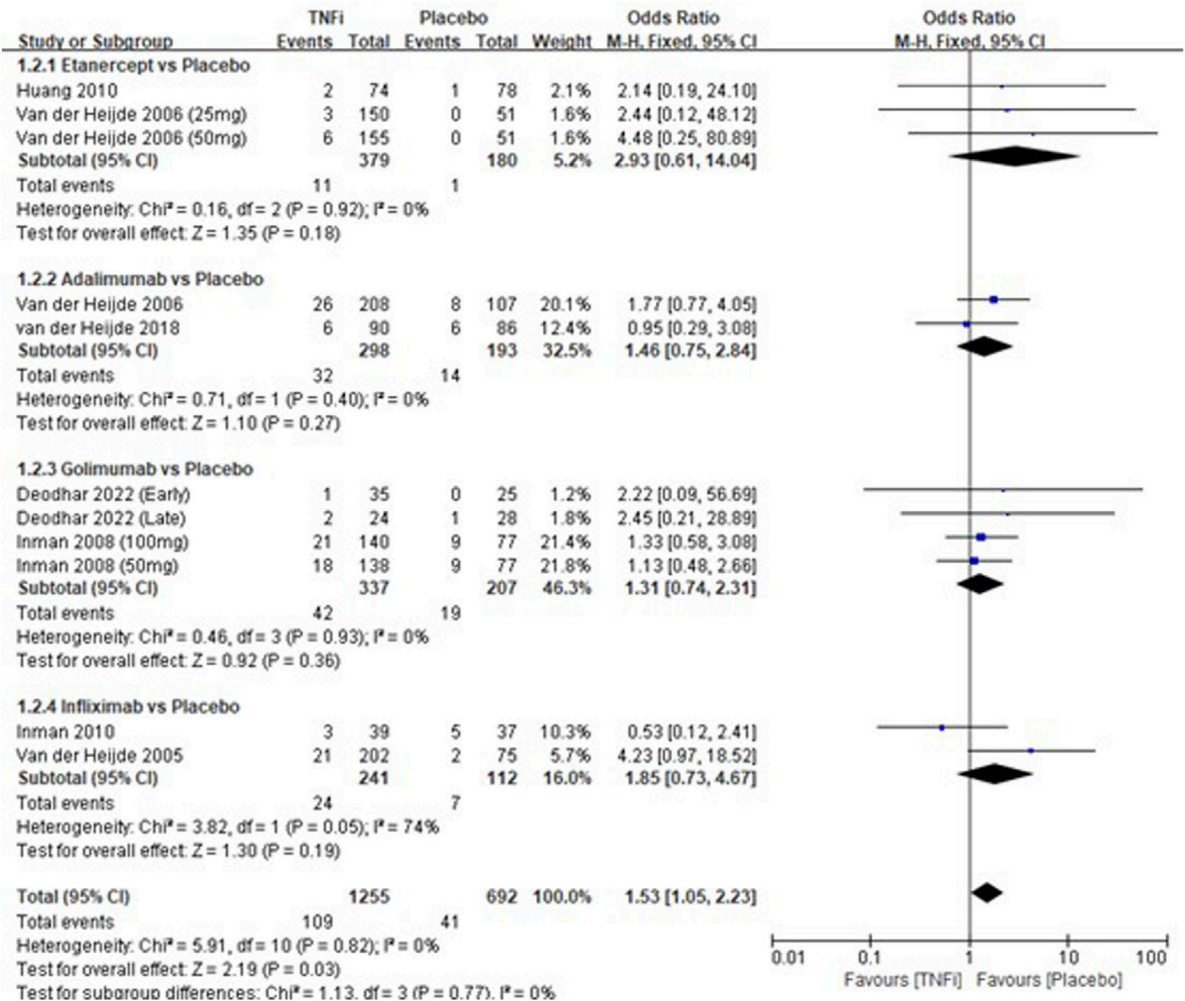
Pooled estimate of the incidence of nasopharyngitis between TNFi and placebo groups in patients with ankylosing spondylitis. The odds ratio >1.0 indicates that the incidence of nasopharyngitis is higher in the TNFi group than that in the placebo group. The subheading “Events” refers to the number of incidences of nasopharyngitis. “Total” refers to the total number of individuals. CI, confidence interval; df, degrees of freedom; M-H, Mantel–Haenszel method of calculation.

A total of 8 studies tested the incidence of nasopharyngitis between TNFi groups and placebo groups for AS treatment. As shown in Figure 3, a meta-analysis of the trials (*n* = 1828) showed a significant difference in the incidence of nasopharyngitis between the two groups (OR = 1.53, 95% CI: 1.05–2.23; *p* = 0.03). However, the subgroup results showed that there was no significant difference in all the subgroups (etanercept *vs* placebo, adalimumab *vs* placebo, golimumab *vs* placebo, and infliximab *vs* placebo) in the incidences of nasopharyngitis.

A total of 4 studies tested the incidence of malignancy between TNFi groups and placebo groups for AS treatment (Inman et al., 2008; Dougados et al., 2010; Huang et al., 2011; Bao et al., 2014). As shown in Supplementary Figure S5, a meta-analysis of the trials (*n* = 650) showed no significant difference in the incidence of malignancy between the two groups (OR = 1.18, 95% CI: 0.34–4.11; *p* = 0.78). The subgroup results showed that there was no significant difference in all subgroups (etanercept *vs* placebo and golimumab *vs* placebo) in the incidences of malignancy events.

**FIGURE 4:**
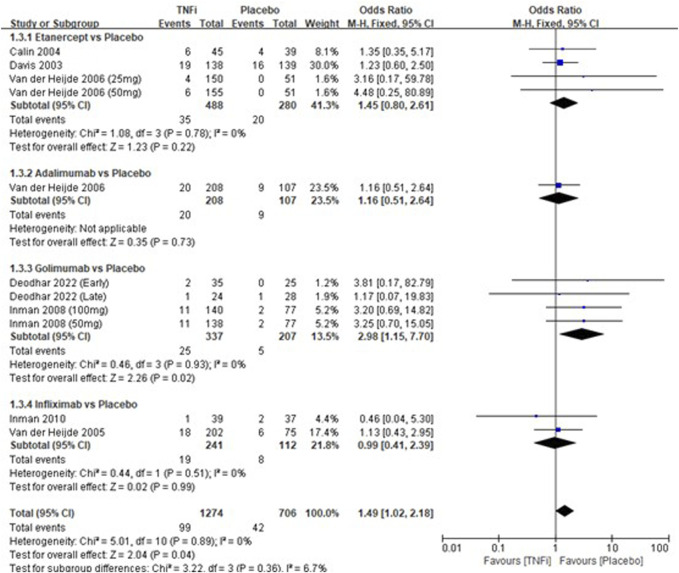
Pooled estimate of the incidence of headache between TNFi and placebo groups in patients with ankylosing spondylitis. The odds ratio >1.0 indicates that the incidence of headache is higher in the TNFi group than that in the placebo group. The subheading “Events” refers to the number of incidences of headache. “Total” refers to the total number of individuals. CI, confidence interval; df, degrees of freedom; M-H, Mantel–Haenszel method of calculation.

A total of 8 studies tested the incidence of headache between TNFi groups and placebo groups for AS treatment. As shown in Figure 4, a meta-analysis of the trials (*n* = 1,852) showed a significant difference in the incidence of headache between the two groups (OR = 1.49, 95% CI: 1.02–2.18; *p* = 0.04). The subgroup results showed that a significant difference in the incidence of headache between golimumab and placebo (OR = 2.98, 95% CI: 1.15–7.70; *p* = 0.02) was found, while no significant differences in the other three subgroups (etanercept *vs* placebo, adalimumab *vs* placebo, and infliximab *vs* placebo) were found in the incidences of headache.

**FIGURE 5:**
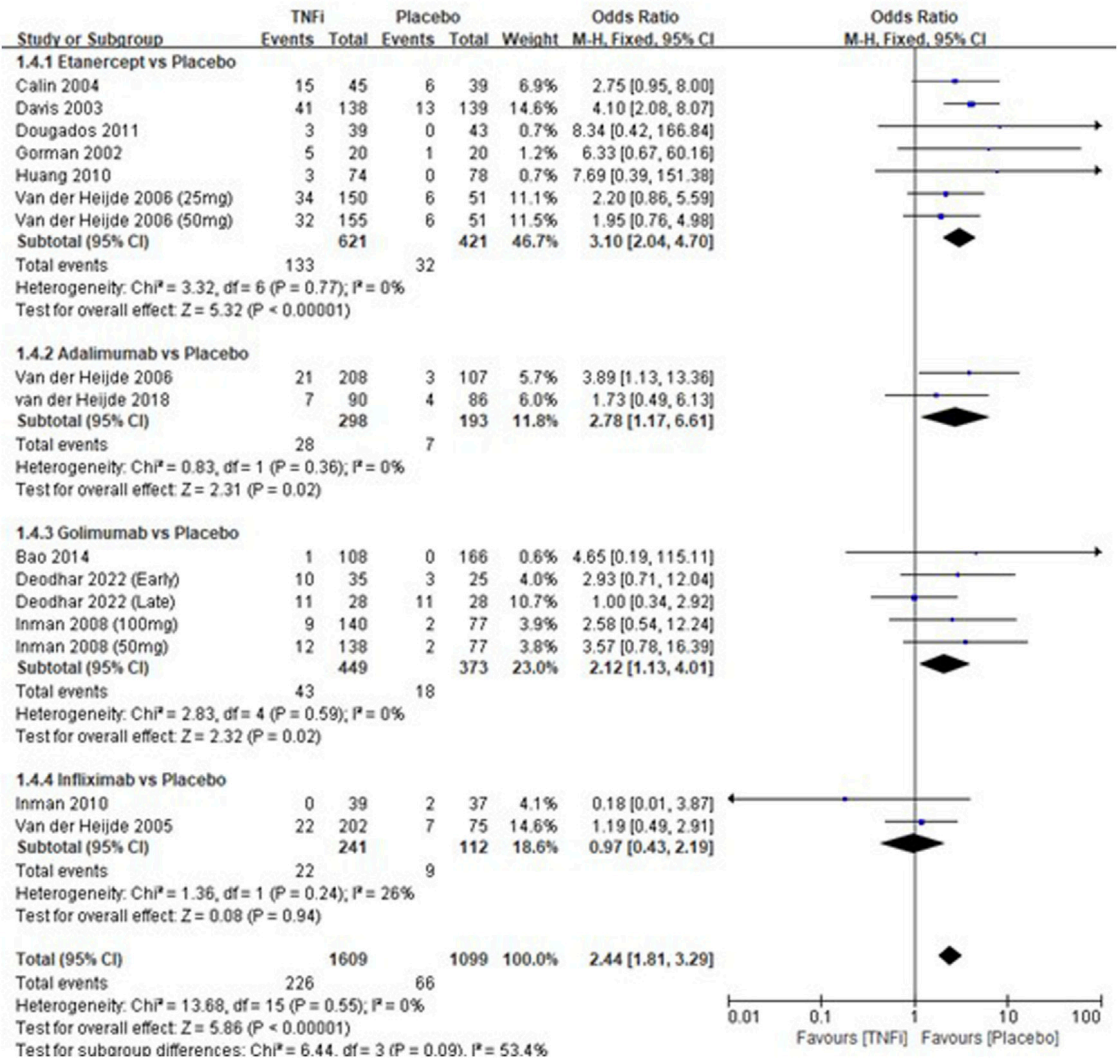
Pooled estimate of the incidence of injection-site reactions between TNFi and placebo groups in patients with ankylosing spondylitis. The odds ratio >1.0 indicates that the incidence of injection-site reactions is higher in the TNFi group than that in the placebo group. The subheading “Events” refers to the number of injection-site reactions. “Total” refers to the total number of individuals. CI, confidence interval; df, degrees of freedom; M-H, Mantel–Haenszel method of calculation.

A total of 12 studies tested the incidence of injection-site reactions between TNFi groups and placebo groups for AS treatment. As shown in Figure 5, a meta-analysis of the trials (*n* = 2,580) showed a significant difference in the incidence of injection-site reactions between TNFi groups and placebo groups (OR = 2.44, 95% CI: 1.81–3.29; *p* < 0.00001). The subgroup results also showed that there were significant differences between etanercept and placebo (OR = 3.10, 95% CI: 2.04–4.70; *p* < 0.00001), between adalimumab and placebo (OR = 2.78, 95% CI: 1.78–6.61; *p* = 0.02), and between golimumab and placebo (OR = 2.12, 95% CI: 1.13–4.01; *p* = 0.02), while no significant difference was found between infliximab and placebo (OR = 0.97, 95% CI: 0.43–2.19; *p* = 0.94) in AS patients.

A total of 7 studies tested the incidence of diarrhea between TNFi groups and placebo groups for AS treatment. As shown in Supplementary Figure S6, a meta-analysis of the trials (*n* = 1,441) showed no significant difference in the incidence of diarrhea between the two groups (OR = 1.25, 95% CI: 0.77–2.01; *p* = 0.36). The subgroup results showed that there is no significant difference in all three subgroups in the incidences of nasopharyngitis.

### 3.5 Publication bias

The publication bias is important for interpreting the conclusions. As shown in Supplementary Figure S7, the funnel plots of the incidence of overall adverse events showed that there was no publication bias.

## 4 Discussion

TNFi (first FDA approval in 2003) has been successfully used for the clinical treatment of AS for two decades, and surprisingly few systematic reports on common adverse events are presently available. This study conducted a meta-analysis of the included studies to comprehensively evaluate the safety of TNFi *vs* the placebo in AS patients. According to the predefined criteria, 18 studies with 3,564 patients were included. The quality assessment showed that the majority of the studies selected had moderate to high methodological quality. This meta-analysis indicated that there was an increased risk of overall adverse events in the TNFi-treated group as compared to the placebo group (OR = 1.55, *p* < 0.0001). Similar to previous meta-analyses (Machado et al., 2013; Liu et al., 2016; Ma H. et al., 2017; Hou et al., 2018), our results also indicated that there was no significant difference in serious adverse events in AS patients (OR = 1.37, *p* = 0.17).

An increasing number of studies showed that TNF is the key mediator of the host response to infection and is indispensable in the process of immune response to many viral infections, suggesting that TNF inhibitors may increase the risk of infections (Germano et al., 2014; Fernández-Ruiz and Aguado, 2018; Singh et al., 2020). The previous meta-analysis on patients with rheumatoid arthritis reported a higher risk of infection and serious infections after TNFi treatment than without TNFi treatment (Bongartz et al., 2006; Michaud et al., 2014). The present study compared the incidences of serious infections, upper respiratory tract infection, and nasopharyngitis in AS patients. Similar to other meta-analyses (Liu et al., 2016; Ma Z. et al., 2017; Xu et al., 2017; Hou et al., 2018), our results showed that there were no significantly increased risks of serious infections in AS patients following TNFi therapies (OR = 1.44, *p* = 0.36). The occurrence rate of upper respiratory tract infection in AS patients treated with TNFi is 14.19%, which is slightly but not significantly increased (OR = 1.22, *p* = 0.16) compared to that treated with the placebo (13.75%). However, the occurrence rate of nasopharyngitis in AS patients treated with TNFi is 8.69%, which is significantly higher (OR = 1.53; *p* = 0.03) than that treated with the placebo (5.67%). With the given small sample sizes and few studies, the subgroup results showed that there was no significant difference in all the subgroups (etanercept *vs* placebo, adalimumab *vs* placebo, golimumab *vs* placebo, and infliximab *vs* placebo) in the incidences of upper respiratory tract infection and nasopharyngitis.

Given the role of TNF in mediating tumor growth, the risk of malignancy with TNFi treatment has been a concern (Pereira et al., 2015). Dougados et al. (2010) reported that one AS patient treated with etanercept was diagnosed with a lung neoplasm after the first injection. Bao et al. (2014) reported that one AS patient treated with golimumab was diagnosed with ovarian cancer after the first injection, which occurred in a 56-year-old woman with a 10-year history of ovarian cysts. Inman et al. (2008) reported that two patients were diagnosed with a malignancy: one in the placebo group and one in the 100-mg golimumab group. In the present meta-analysis, there was no statistical difference in the incidences of malignancies between TNFi groups and placebo groups (OR = 1.18; *p* = 0.79).

It is interesting to note that the occurrence rate of headache in AS patients treated with TNFi is 7.77%, which is statistically higher (OR = 1.49; *p* = 0.04) than that treated with the placebo (5.95%). Subgroup analysis showed that golimumab is significantly associated with a markedly increased risk of headache (OR = 2.98, *p* = 0.02). Traditionally, the pro-inflammatory cytokine TNF-α plays a role in migraine pathophysiology (Kraig et al., 2010; Rainero et al., 2014). However, Rozen and Swidan (2007) found that cerebrospinal fluid TNF-α levels were high, but serum TNF-α levels were normal in patients with new daily persistent headache. TNFi are the macromolecules that cannot cross the blood–brain barrier; they could not reflect the levels of TNF-α in cerebrospinal fluid (Pardridge, 2010). Therefore, golimumab treatment is able to alleviate back pain symptoms, but it may also attract patient’s attention in alleviating symptoms from back pain to headache.

Injection-site reactions are a major complication for all FDA-approved injectable biological agents (Thomaidou and Ramot, 2019). The previous meta-analysis on AS patients reported a higher risk of injection-site reactions after TNFi treatment than without TNFi treatment (Ma H. et al., 2017). Similar to the previous meta-analyses, the occurrence rate of injection-site reactions in AS patients treated with TNFi is 14.05%, which is markedly higher (OR = 2.44, *p* < 0.00001) than that treated with the placebo (5.98%). However, no significant difference was found between infliximab and placebo in the incidences of injection-site reactions, which may be due to the insufficient sample size.

The results indicated that there was an increased risk of overall adverse events in the TNFi-treated group compared to the placebo group. However, there was no significant difference in serious adverse events, malignancy events, and upper respiratory tract infection in AS patients between TNFi and placebo groups. It was probably due to the increased risk of common adverse events after TNFi treatment (i.e., injection-site reactions, nasopharyngitis, and headache), and these adverse events are the probable factors affecting medication compliance and persistence.

Given that there is no randomized placebo-controlled trial comparing certolizumab pegol with the placebo in patients with only AS, the other four TNFi (etanercept, adalimumab, golimumab, and infliximab) were included in the present study. Landewé et al. (2014) reported the similar adverse events between TNFi and placebo in patients with AS or non-radiographic axial spondyloarthritis, and the most common adverse events were nasopharyngitis and upper respiratory tract infection. In addition, Babuna Kobaner et al. (2018) presented a case treated with certolizumab pegol that induced a generalized psoriasiform eruption in an AS patient.

Finally, this study still has some limitations, which should be addressed. First, the present study was not able to compare the exact incidence of “serious adverse events” and “serious infections” in AS patients, due to the incongruent definition across studies and the small number of patients with serious adverse events or serious infections. Thus, it was unclear on the risk of bias for this domain in those studies. Second, all included studies were of fairly short duration (6–24 weeks), with a median duration of 12 weeks. Further research is needed to evaluate the long-term safety. Third, the type of placebo was not stated in detail for most of the included studies; different placebos probably have different mechanisms of action that could in turn influence the outcomes. Fourth, as this study analyzed only the adverse events in patients treated with TNFi compared with the placebo group, further research is needed to examine the adverse events among the five TNFi.

## 5 Conclusion

The present meta-analysis indicated that there was no significant difference in serious adverse events, serious infections, upper respiratory tract infection, malignancies, and diarrhea in AS patients between TNFi and placebo groups. However, the patients who received TNFi experienced more injection-site reactions, nasopharyngitis, and headache than those who received the placebo. Considering the limitations of the included studies, large-scale and long-term follow-up clinical trials are expected to further quantify the safety of TNFi in AS treatment.

## Data Availability

The original contributions presented in the study are included in the article/Supplementary Material; further inquiries can be directed to the corresponding author.

## Supplementary material

SUPPLEMENTARY FIGURE S1 Methodological quality assessment of trials using the Cochrane risk of bias tool. The symbols show a low risk of bias (+) or unclear risk of bias (?).

SUPPLEMENTARY FIGURE S2 Pooled estimate of the incidence of serious adverse events between TNFi groups and placebo groups in patients with ankylosing spondylitis. The odds ratio >1.0 indicates that the incidence of serious adverse events is higher in the TNFi group than that in the placebo group. The subheading “Events” refers to the number of serious adverse events. “Total” refers to the total number of individuals. CI, confidence interval; df, degrees of freedom; M-H, Mantel–Haenszel method of calculation.

SUPPLEMENTARY FIGURE S3 Pooled estimate of the incidence of serious infections between TNFi groups and placebo groups in patients with ankylosing spondylitis. The odds ratio >1.0 indicates that the incidence of serious infections is higher in the TNFi group than that in the placebo group. The subheading “Events” refers to the number of serious infections. “Total” refers to the total number of individuals. CI, confidence interval; df, degrees of freedom; M-H, Mantel–Haenszel method of calculation.

SUPPLEMENTARY FIGURE S4 Pooled estimate of the incidence of upper respiratory tract infection between TNFi and placebo groups in patients with ankylosing spondylitis. The odds ratio >1.0 indicates that the incidence of upper respiratory tract infection is higher in the TNFi group than that in the placebo group. The subheading “Events” refers to the number of upper respiratory tract infections. “Total” refers to the total number of individuals. CI, confidence interval; df, degrees of freedom; M-H, Mantel–Haenszel method of calculation.

SUPPLEMENTARY FIGURE S5 Pooled estimate of the incidence of malignancy events between TNFi and placebo groups in patients with ankylosing spondylitis. The odds ratio >1.0 indicates that the incidence of malignancy events is higher in the TNFi group than that in the placebo group. The subheading “Events” refers to the number of malignancy events. “Total” refers to the total number of individuals. CI, confidence interval; df, degrees of freedom; M-H, Mantel–Haenszel method of calculation.

SUPPLEMENTARY FIGURE S6 Pooled estimate of the incidence of diarrhea between TNFi and placebo groups in patients with ankylosing spondylitis. The odds ratio > 1.0 indicates that the incidence of diarrhea is higher in the TNFi group than that in the placebo group. The subheading “Events” refers to the number of incidences of diarrhea. “Total” refers to the total number of individuals. CI, confidence interval; df, degrees of freedom; M-H, Mantel–Haenszel method of calculation.

SUPPLEMENTARY FIGURE S7 Funnel plots of randomized controlled trials.
