# Supplementary figures and images for: Adverse events of tumor necrosis factor alpha inhibitors for the treatment of ankylosing spondylitis: A meta-analysis of randomized, placebo-controlled trials
Source: Front Pharmacol. 2023 Feb 13;14:1084614. doi: 10.3389/fphar.2023.1084614 (PMC9972296; doi:10.3389/fphar.2023.1084614)

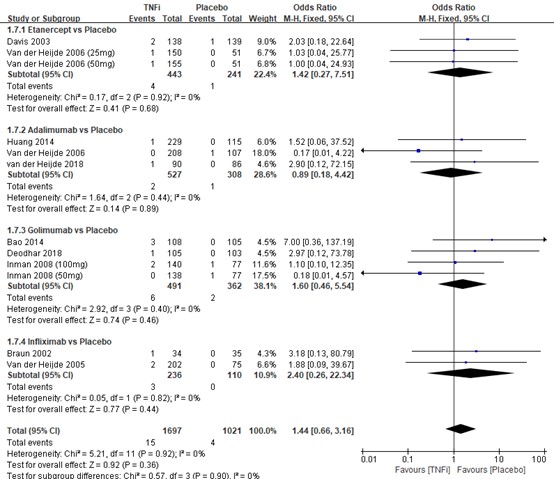

Supplement: Supplementary file 1 [file Image3.JPEG]

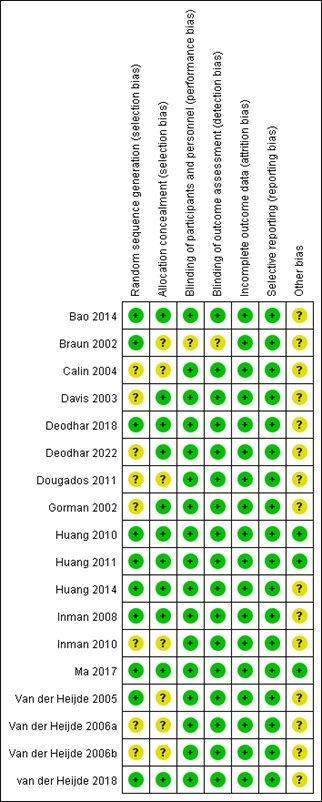

Supplement: Supplementary file 2 [file Image1.JPEG]

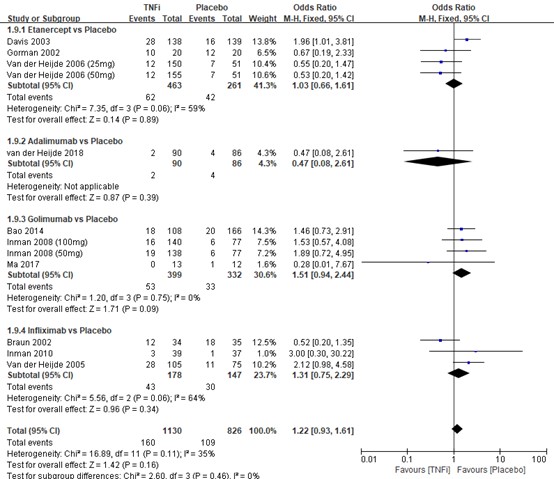

Supplement: Supplementary file 3 [file Image4.JPEG]

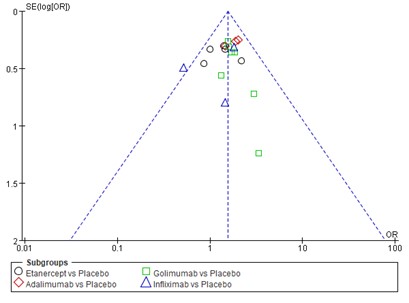

Supplement: Supplementary file 4 [file Image7.JPEG]

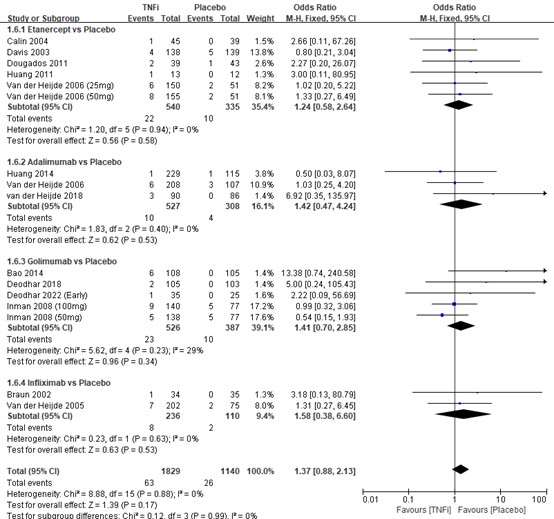

Supplement: Supplementary file 5 [file Image2.JPEG]

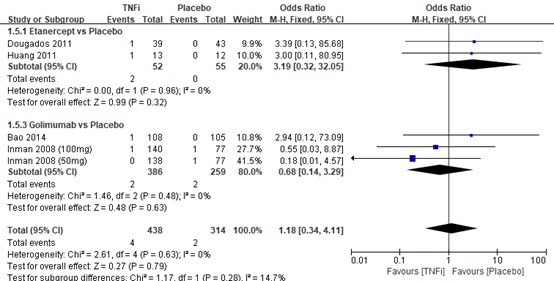

Supplement: Supplementary file 6 [file Image5.JPEG]

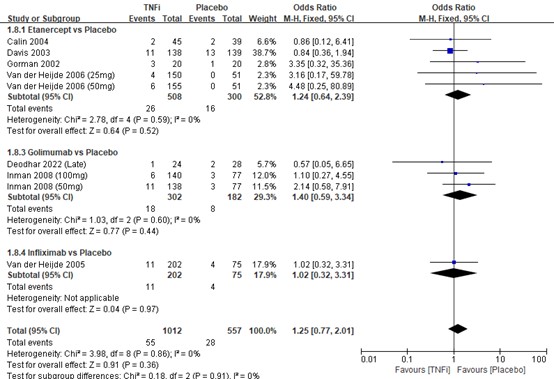

Supplement: Supplementary file 7 [file Image6.JPEG]
